# Altered CO_2_ sensitivity of connexin26 mutant hemichannels *in vitro*

**DOI:** 10.14814/phy2.13038

**Published:** 2016-11-24

**Authors:** Elizabeth de Wolf, Joseph van de Wiel, Jonathan Cook, Nicholas Dale

**Affiliations:** ^1^ School of Life SciencesUniversity of WarwickCoventryUnited Kingdom

**Keywords:** Connexin, hemichannel, non‐syndromic hearing loss, respiratory chemosensitivity, syndromic hearing loss

## Abstract

Connexin26 (Cx26) mutations underlie human pathologies ranging from hearing loss to keratitis ichthyosis deafness (KID) syndrome. Cx26 hemichannels are directly gated by CO_2_ and contribute to the chemosensory regulation of breathing. The KID syndrome mutation A88V is insensitive to CO_2_, and has a dominant negative action on the CO_2_ sensitivity of Cx26^WT^ hemichannels, and reduces respiratory drive in humans. We have now examined the effect of further human mutations of Cx26 on its sensitivity to CO_2_ **.** Mutated Cx26 subunits, carrying one of A88S, N14K, N14Y, M34T, or V84L, were transiently expressed in HeLa cells. The CO_2_‐dependence of hemichannel activity, and their ability to exert dominant negative actions on cells stably expressing Cx26^WT^, was quantified by a dye‐loading assay. The KID syndrome mutation, N14K, abolished the sensitivity of Cx26 to CO_2_. Both N14Y and N14K exerted a powerful dominant negative action on the CO_2_ sensitivity of Cx26^WT^. None of the other mutations (all recessive) had a dominant negative action. A88S shifted the affinity of Cx26 to slightly higher levels without reducing its ability to fully open to CO_2_. M34T did not change the affinity of Cx26 for CO_2_ but reduced its ability to open in response to CO_2_. V84L had no effect on the CO_2_‐sensitivity of Cx26. Some pathological mutations of Cx26 can therefore alter the CO_2_ sensitivity of Cx26 hemichannels. The loss of CO_2_ sensitivity could contribute to pathology and consequent reduced respiratory drive could be an unrecognized comorbidity of these pathologies.

## Introduction

Connexins are a family of intrinsic membrane proteins that can form gap junctions, which enable intercellular transfer of ions and small molecules. In a gap junction, the hexameric channels or connexons in each opposed membrane dock together to form an intercellular channel. In addition to this canonical role of connexins, unopposed connexin hexamers or hemichannels can also mediate intercellular signaling (Kamermans et al. 2001; Stout et al. 2002; Goodenough and Paul 2003; Saez et al. 2003; Stout and Charles 2003; Pearson et al. 2005; Huckstepp et al. 2010a,b). Hemichannels are capable of opening in response to a variety of stimuli. The release of ATP via open hemichannels can signal to other cells via a range of ionotropic and metabotropic ATP receptors. We have previously demonstrated that connexin26 (Cx26) hemichannels are opened directly by an increase in PCO_2_, allowing the release of ATP, which acts on respiratory neurons to stimulate breathing (Huckstepp et al. 2010a,b). Mutational analysis has demonstrated the direct action of CO_2_ on the Cx26 hemichannel via the carbamylation of K125. This carbamylated residue can form a salt bridge to R104 in an adjacent subunit of the connexin hexamer (Meigh et al. 2013).

Mutations in Cx26 are linked to human pathology, ranging from non‐syndromic sensorineural hearing loss (NSHL) to more severe syndromic hearing loss, including keratitis ichthyosis deafness (KID) syndrome (Xu and Nicholson 2013). KID syndrome is characterized by skin lesions, sensorineural hearing loss, and vascularizing keratitis. To date, nine mutations have been linked to KID syndrome. These are dominant missense mutations that permit the formation of functional Cx26 gap junctions and hemichannels with altered properties (van Geel et al. 2002; Yotsumoto et al. 2003; Sonoda et al. 2004; Janecke et al. 2005; Mazereeuw‐Hautier et al. 2007; Kelly et al. 2008; Lazic et al. 2008; Arndt et al. 2010; Koppelhus et al. 2010; Sbidian et al. 2010; Terrinoni et al. 2010a,b; de Zwart‐Storm et al. 2011).

It is curious that deletion of Cx26 (e.g., through the 35delG mutation which introduces a premature stop codon (Kelley et al. 1998)) causes profound deafness but does not result in the devastating pathologies characteristic of KID syndrome in either human or rodent models (Sun et al. 2009; Wang et al. 2009). This has led to the suggestion that the syndromic pathologies arise from a gain of function caused by the KID syndrome mutations. One common suggestion is that the Cx26 hemichannels carrying the KID syndrome mutations are leaky, and thus cause cell death (Gerido et al. 2007; Mhaske et al. 2013).

The effects of human disease‐causing mutations on the CO_2_ sensitivity of Cx26 have not been extensively explored. Recently we reported that the KID syndrome mutation A88V abolished the sensitivity of Cx26 to CO_2_ (Meigh et al. 2014). This mutation acted in a dominant fashion to abolish the CO_2_ sensitivity of wild‐type Cx26 hemichannels (Meigh et al. 2014). Interestingly, the patient carrying this mutation experienced central apnea and disordered breathing (Meigh et al. 2014).

In this paper, we explore the extent to which other Cx26 mutations associated with varying degrees of human pathology alter the CO_2_ sensitivity of Cx26. We have selected a range of mutations to include one which occurs at the same A88 residue of our prior work, but does not cause pathology (A88S), two further KID syndrome mutations (N14K and N14Y), and two recessive mutations that cause non‐syndromic deafness (M34T and V84L). We found that the effects of these mutations on the CO_2_ sensitivity of Cx26 broadly correlated with the severity of human pathology caused by these mutations.

## Methods

### Connexin 26 mutagenesis

Mutant Cx26 was produced from wild‐type Cx26 in the Puc19 vector via the Quikchange protocol using the following primers: Cx26^N14K^, forward 5′ ATC CTC GGG GGT GTC AAG AAG CAC TCC ACC AGC 3′, reverse 5′ GCT GGT GGA GTG CTT CTT GAC ACC CCC GAG GAT 3′; Cx26^N14Y^, forward 5′ ATC CTC GGG GGT GTC TAC AAG CAC TCC ACC AGC 3′, reverse 5′ GCT GGT GGA GTG CTT GTA GAC ACC CCC GAG GAT 3′; Cx26^A88S^, forward 5′ G GTG TCC ACG CCG AGC CTC CTG GTA GCT 3′, reverse 5′ AGC TAC CAG GAG GCT CGG CGT GGA CAC C 3′; Cx26^M34T^, forward 5′ TC ATC TTC CGC ATC ACG ATC CTC GTG GTG GC 3′, reverse 5′ GC CAC CAC GAG GAT CGT GAT GCG GAA GAT GA 3′; Cx26^V84L^, forward 5′ G CAG CTG ATC ATG CTG TCC ACG CCG GC 3′, reverse 5′ GC CGG CGT GGA CAG CAT GAT CAG CTG C 3′. The sequence for wild‐type Cx26 was taken from accession number: NM_001004099.1. Each mutant Cx26 was subcloned into the pCAG‐GS mCherry vector prior to mammalian cell transfection. This vector expressed Cx26 as a fusion protein with mCherry. The presence of Cx26 containing only the required mutation was confirmed by DNA sequencing (GATC Biotech).

### HeLa cell culture

HeLa cells were grown in Dulbecco's Modified Eagle Medium (DMEM) supplemented with 10% fetal bovine serum, 50 *μ*g/mL penicillin/streptomycin, and 3 mmol/L CaCl_2_. For dye loading experiments, cells were plated onto coverslips at a density of 5 × 10^4^ cells per well, or 1 × 10^4^ cells per well for HeLa cells stably expressing Cx26^WT^. HeLa cells were transiently transfected with Cx26 constructs as described in the GeneJuice Transfection Reagent protocol (Novagen, Millipore (UK) Ltd, Watford, UK).

### aCSF solutions

#### Control aCSF

124 mmol/L NaCl, 26 mmol/L NaHCO_3_, 1.25 mmol/L NaH_2_PO_4_, 3 mmol/L KCl, 10 mmol/L d‐glucose, 1 mmol/L MgSO_4_, 2 mmol/L CaCl_2_, bubbled with 95%O_2_ 5% CO_2_ with a final pH of ~7.4. This Artificial cerebrospinal fluid (aCSF) has a PCO_2_ of 35 mmHg.

#### Zero Ca^2+^ aCSF

124 mmol/L NaCl, 26 mmol/L NaHCO_3_, 1.25 mmol/L NaH_2_PO_4_, 3 mmol/L KCl, 10 mmol/L d‐glucose, 1 mmol/L MgSO_4_, 2 mmol/L MgCl_2_, 1 mmol/L EGTA, bubbled with 95%O_2_ 5% CO_2_ with a final pH of ~7.4.

#### Hypercapnic (55 mmHg CO_2_) aCSF

100 mmol/L NaCl, 50 mmol/L NaHCO_3_, 1.25 mmol/L NaH_2_PO_4_, 3 mmol/L KCl, 10 mmol/L d‐glucose, 1 mmol/L MgSO_4_, 2 mmol/L CaCl_2_.

#### Hypercapnic (70 mmHg CO_2_) aCSF

70 mmol/L NaCl, 80 mmol/L NaHCO_3_, 1.25 mmol/L NaH_2_PO_4_, 3 mmol/L KCl, 10 mmol/L d‐glucose, 1 mmol/L MgSO_4_, 2 mmol/L CaCl_2_.

Hypercapnic aCSF was saturated with sufficient CO_2_ (the remaining balance being O_2_) to adjust the final pH to approximately the same as that of the control aCSF (~pH 7.4), thereby minimizing the potential effects of changes in extracellular pH.

### Dye loading experiments

HeLa cells expressing wild type and mutant Cx26 for 3 or 6 days were exposed to hypercapnic aCSF (55 mmHg or 70 mmHg CO_2_) containing 200 *μ*mol/L 5(6)‐carboxyfluorescein (CBF) for 10 min. Subsequently, cells were exposed to control aCSF with 200 *μ*mol/L CBF for 5 min, followed by a wash control aCSF without CBF for 30 min to remove excess extracellular dye.

Baseline dye loading was determined by exposing HeLa cells to 200 *μ*mol/L CBF in control aCSF for 15 min, followed by a 30 min wash in control aCSF.

A zero Ca^2+^‐positive control was undertaken to verify the presence of functional connexin hemichannels. HeLa cells were exposed to 200 *μ*mol/L CBF in zero Ca^2+^ aCSF for 10 min, followed by control aCSF with 200 *μ*mol/L CBF for 5 min and 30 min of washing with aCSF.

### Connexin 26 immunostaining

**Figure 1:**
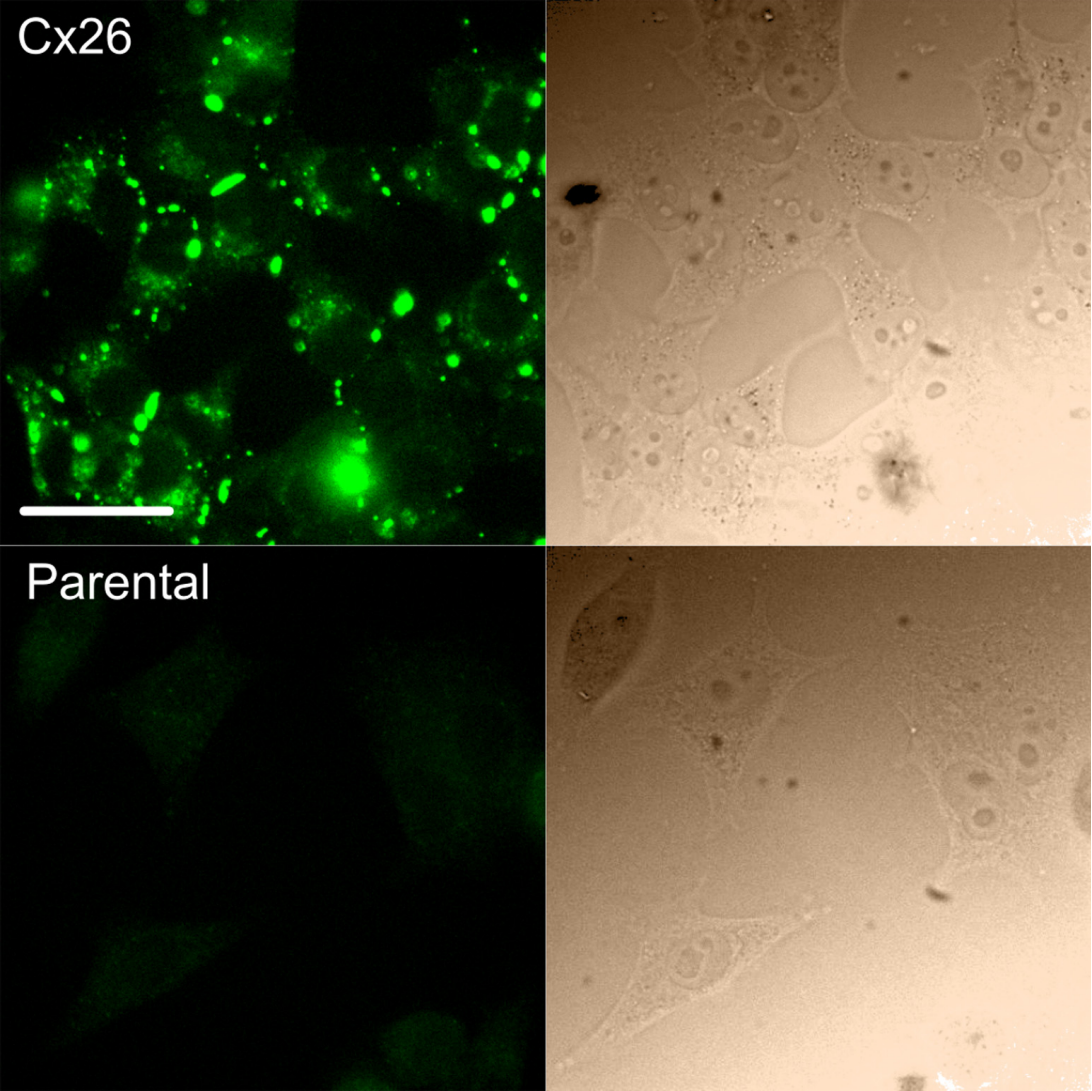
Expression of Cx26 in HeLa cells. Top row, immunofluorescent staining for Cx26 in HeLa cells stably expressing Cx26. This demonstrates abundant expression of fluorescent puncta representing Cx26 hemichannels and gap junctions. Bottom row, parental HeLa cells do not exhibit any immunofluorescent puncta. In both rows, the corresponding bright‐field image is shown on the right. Scale bar 40 *μ*m.

To confirm the presence of Cx26 in HeLa cells stably expressing Cx26 (Fig 1), cells were fixed, permeabilized and stained with antibody (mouse anti‐Connexin‐26 antibody, (ThermoFisher Scientific, Life Technologies Ltd, Paisley, UK), #138100, 1/500 overnight at 4°C; followed by AlexaFluor 488 anti‐mouse IgG, Life Technologies, #A21202, 1/1000 for 2 h at room temperature) as previously described (Huckstepp et al. 2010a).

### Fluorescence imaging and statistical analysis

**Figure 2:**
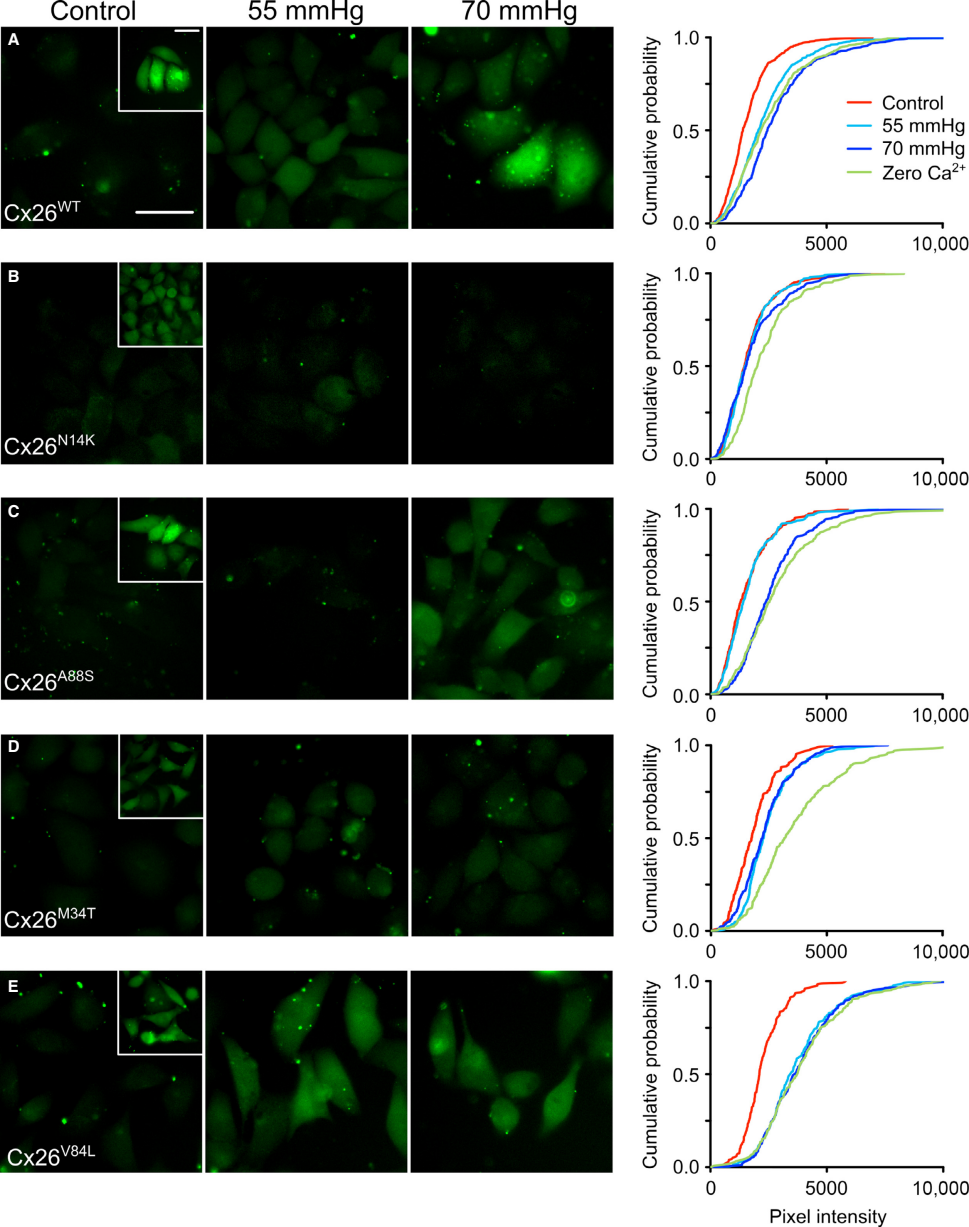
CO_2_‐dependent dye loading in wild‐type Cx26 and mutants. Representative images showing the baseline dye loading under control conditions (Control) and two values of elevated PCO_2_ (55 mHg and 70 mmHg). The insets show representative images of dye loading evoked by exposure to zero Ca^2+^. Scale bars 40 *μ*m. The cumulative probability distributions for each variant of Cx26 are shown on the right and comprise measurements from at least five independent replicates (50 measurements per replicate).

Following dye loading and immunostaining, HeLa cells were imaged by epifluorescence (Scientifica Slice Scope, Cairn Research OptoLED illumination, 60 × water Olympus immersion objective, NA 1.0, Hamamatsu ImagEM EM‐CCD camera, Metafluor software). To further confirm the presence of the Cx26 construct, the mCherry fluorescent protein tag was also imaged by epifluorescence. ImageJ (Schneider et al. 2012) was used to measure the extent of dye loading by drawing a region of interest (ROI) around each cell, and subsequently, the mean pixel intensity of the ROI was determined. The mean pixel intensity of a representative background ROI for each image was subtracted from each cell measurement from the same image. At least 50 cells were measured for each condition per experiment, and at least five independent repetitions using the same batch of HeLa cells (ATCC) were completed. The mean pixel intensities were plotted as cumulative probability distributions in Figures 2 and 4 and these show every data point measured.

To avoid pseudoreplication, statistical analysis was performed on the medians of the independent replicates. The median differences between the control, hypercapnic dye loading, and zero Ca^2+^ dye loading were assessed by nonparametric statistical tests (Kruskal–Wallis ANOVA for multiple comparisons and Mann–Whitney test for pairwise comparisons). Data are presented as the median, with the error bars being the upper and lower quartiles.

## Results

We evaluated the CO_2_ sensitivity of wild type and mutant Cx26 hemichannels via our previously established and validated dye‐loading assay (Huckstepp et al. 2010a; Meigh et al. 2013, 2014, 2015). The parental HeLa cells do not load with dye in response to either a CO_2_ stimulus or removal of extracellular Ca^2+^ following transfection with an empty vector (Meigh et al. 2013, 2014, 2015). The parental HeLa cells thus have no endogenous hemichannels or CO_2_ sensitivity and are an ideal expression system for Cx26 and its mutant subunits. Three days after transient transfection, we tested the HeLa cells expressing the Cx26 subunits for CO_2_ sensitivity and hemichannel function.

### The effect of pathological mutations on the sensitivity of Cx26 hemichannels to CO_2_

**Figure 3:**
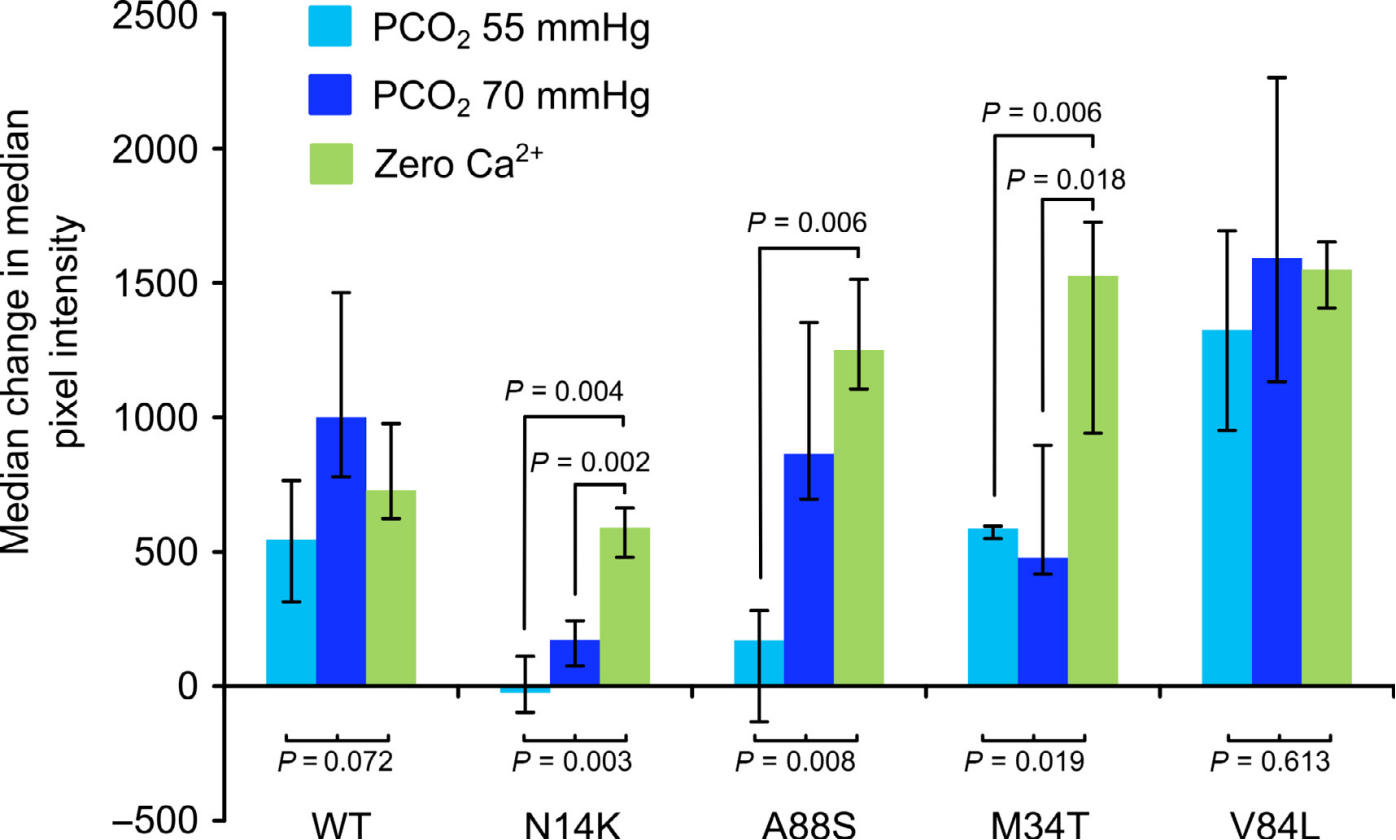
Summary of CO_2_ sensitivity of wild‐type Cx26 and mutants. The comparisons under the graph are from the Kruskal–Wallis ANOVA. Pairwise comparisons made with Mann–Whitney U test. A88S shifts CO_2_ sensitivity to higher levels of PCO_2_, N14K abolishes CO_2_ sensitivity, M34T substantially reduces the dye loading during the CO_2_ challenge, and V84L has no effect. Error bars are the first and third quartiles.

As expected, HeLa cells expressing Cx26^WT^ loaded with dye in a CO_2_‐dependent manner that graded with the doses of CO_2_ (55 and 70 mmHg, Figs. 2A and 3) that reflect mild to moderate hypercapnia (Huckstepp et al. 2010b). We used a further stimulus, removal of extracellular Ca^2+^, as a mechanistically independent assay of hemichannel function. The dye loading in zero Ca^2+^ acts as a positive control and indicates the maximal extent of possible dye loading through the open hemichannel (Sanchez et al. 2010).

**Figure 4:**
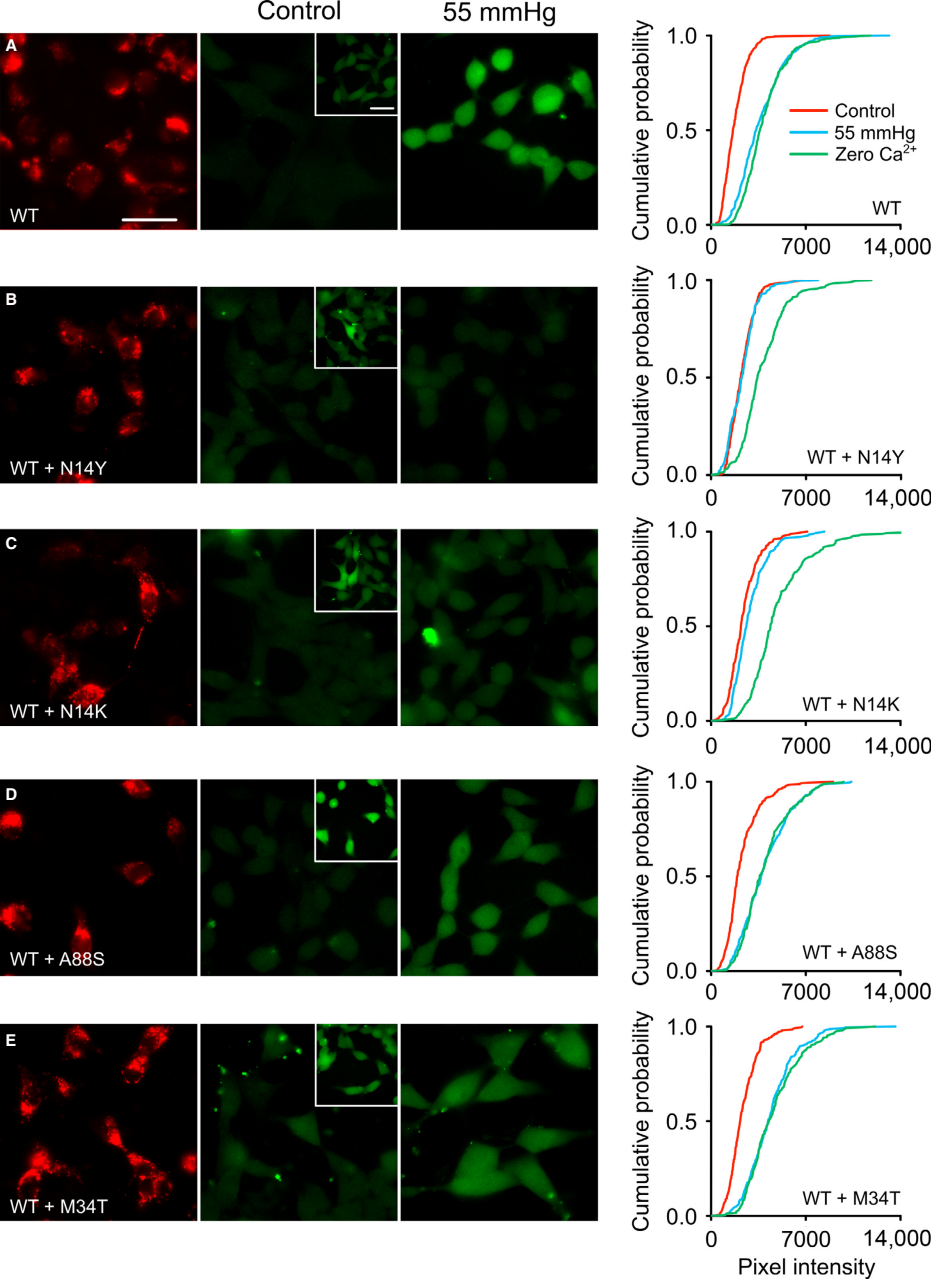
Effects of expressing mutant Cx26 subunits in HeLa cells that stably express Cx26^WT^. The first column shows the mCherry fluorescence tagged to the Cx26 subunits to demonstrate expression of the mutant subunits at 6 days following transfection. CO_2_‐dependent dye loading was assessed 6 days after transfection of the mutant subunits. Only the N14K and N14Y subunits have a dominant negative action. The insets show dye loading in response to the positive control zero Ca^2+^ stimulus. Scale bars 40 *μ*m. The cumulative probability distributions comprise measurements from at least five independent replicates (50 measurements per replicate).

In parental HeLa cells, we found the KID syndrome mutations hard to express – there was a tendency for cells to die, and thus the level of expression of the mCherry tag in living cells was low. Nevertheless, Cx26^N14K^ hemichannels were able to express sufficiently to demonstrate that the positive control zero Ca^2+^ stimulus resulted in dye loading that was significantly above the control (Figs. 2B, 3). Thus, functional Cx26^N14K^ hemichannels were present in the plasma membrane. However, HeLa cells expressing Cx26^N14K^ showed no dye loading to CO_2_ at either 55 mmHg or 70 mmHg. This mutation therefore abolishes the sensitivity of Cx26 to CO_2_. We were unable to express Cx26^N14Y^ in parental HeLa cells, however, it was possible to express both the Cx26^N14Y^ subunits in HeLa cells that stably expressed Cx26^WT^ (Fig. 4B and discussed later).

The mutation A88S altered the sensitivity of Cx26 hemichannels to CO_2_. Although no dye loading was seen in response to a PCO_2_ of 55 mmHg, full dye loading, indistinguishable from the zero Ca^2+^ stimulus, was seen in response to the higher PCO_2_ stimulus of 70 mmHg (Figs. 2C and 3). Thus, this mutation does not abolish CO_2_ sensitivity, but rather shifts it toward slightly higher levels of PCO_2_.

HeLa cells expressing Cx26^M34T^ showed reduced capacity for dye loading in response to both CO_2_ stimuli (Figs. 2D and 3). Nevertheless, the zero Ca^2+^ stimulus gave robust and much greater dye loading. This suggests that while the affinity of CO_2_ for the Cx26 hemichannel had not been markedly altered by this mutation, the capacity of the hemichannel to open in response to CO_2_ and admit the fluorescent dye had been reduced. Finally, the mutation V84L had no effect on the sensitivity of Cx26 to CO_2_. HeLa cells that expressed Cx26^V84L^ robustly loaded with dye at both levels of PCO_2_ (Figs. 2E and 3).

### Only dominant Cx26 mutations have a dominant negative effect on CO_2_ sensitivity

A notable feature of the syndromic mutations of Cx26 is that they are dominant and for KID syndrome have only been reported in patients as heterozygous. We previously demonstrated the dominant negative action of A88V on the CO_2_ sensitivity of Cx26^WT^. We found that the dominant negative action of Cx26^A88V^ took 6 days to manifest itself (Meigh et al. 2014). Therefore, using the same assay, we transfected HeLa cells stably expressing nontagged Cx26^WT^ with mCherry‐tagged mutant subunits and evaluated their CO_2_ sensitivity 6 days after transfection (Fig. 4). The mCherry fluorescence showed that after 6 days, there was abundant expression of all of the mutated subunits (Fig. 4).

**Figure 5:**
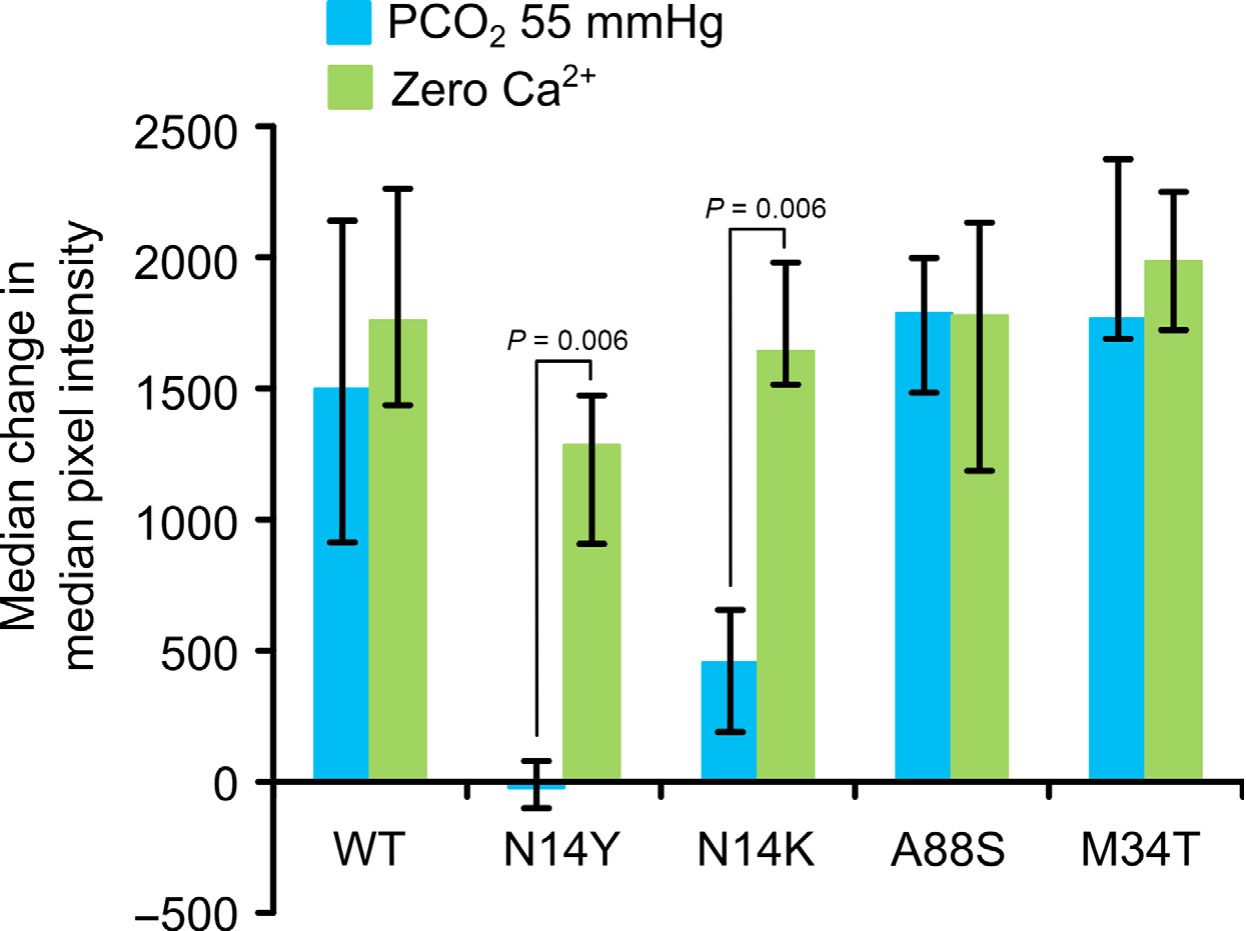
Summary of the actions of mutant Cx26 subunits on dye loading in HeLa cells stably expressing Cx26^WT^. The positive control for dye loading is that occurring under conditions of zero Ca^2+^. Only in the case of N14K and N14Y is there a reduction in the CO_2_‐dependent dye loading relative to that of zero Ca^2+^, indicating a very strong dominant negative action of these mutant subunits on the CO_2_ sensitivity of wild‐type Cx26. Error bars are the first and third quartiles.

As stated earlier, we found that Cx26^WT^ HeLa cells retained their sensitivity to CO_2_ over this 6 day period (Fig. 4A). However, the Cx26^N14K^ and Cx26^N14Y^ subunits had a powerful dominant negative action. Coexpression of these subunits greatly reduced the CO_2_‐sensitivity of HeLa cells expressing Cx26^WT^. Nevertheless, functional hemichannels were still present as the zero Ca^2+^ control gave robust dye loading (Figs. 4B,C and 5).

Interestingly, despite being strongly expressed, neither A88S nor M34T had any dominant negative action. HeLa cells expressing Cx26^WT^ completely retained their CO_2_ sensitivity when transfected with either Cx26^A88S^ or Cx26^M34T^ (Figs. 4D,E and 5).

## Discussion

### Potential limitations to the study

A reviewer has asked whether this study was performed blind to mutation to avoid introducing bias. We did not perform these studies in a blinded fashion. While blinding is always desirable, in practice it would have been rather tricky to achieve with the available resources. To avoid selection bias, we measured many fields from the cover slips of cells and obtained measurements from at least 50 cells per coverslip. The selection of fields for analysis was guided by the mCherry expression (i.e., those cells in which the connexin is expressed). Given that we had no prior knowledge or hypothesis as to what the mutations should do to CO_2_ sensitivity, and that we have seen all possible outcomes with respect to effects on CO_2_ sensitivity for the various mutations, we believe our assay to be unbiased. In our prior studies, the dye loading results obtained using the methods outlined here correspond very closely to electrophysiological measurements of hemichannel activity (Huckstepp et al. 2010a; Meigh et al. 2013, 2015), giving us further reassurance that our measurements were unbiased.

### KID syndrome and CO_2_ sensitivity

We have advanced our previous work by elucidating the effect of a wider range of pathological mutations on the CO_2_ sensitivity of Cx26 hemichannels (Table 1). We previously documented that the A88V mutation, linked to a severe and fatal form of KID syndrome, abolished the sensitivity of Cx26 to CO_2_ in a dominant negative fashion (Meigh et al. 2014). Intriguingly, we now find that a different mutation at the same residue, A88S, had a rather modest effect on CO_2_ sensitivity, which was shifted to slightly higher values of PCO_2_. Furthermore Cx26^A88S^ had no dominant action on the CO_2_ sensitivity of Cx26^WT^. This matches the effects of Cx26^A88S^ on human physiology: this mutation is non‐syndromic, recessive, and is not monogenically linked to deafness (Frei et al. 2002). No homozygotes for A88S have been reported in the literature to date. The contrast between these two different mutations at the same locus (A88V and A88S) supports our hypothesis that the dominant negative action on the CO_2_ sensitivity of the Cx26 hemichannel may be an underlying mechanistic contributor to human pathology.

**Table 1:** Summary of effects of Cx26 mutations on the CO_2_ sensitivity of Cx26 hemichannels and human pathology

	CO_2_ actions on hemichannel		Human pathology	
	Sensitivity of mutant hemichannel	DN action on Cx26^WT^ hemichannel	Phenotype	Dominant or recessive
A88V	Abolished (Meigh et al. 2014)	Yes −100% (Meigh et al. 2014)	KIDS – die in infancy (Koppelhus et al. 2010)	Dominant (Koppelhus et al. 2010)
N14Y	Not tested	Yes −100%	KIDS – survive to childhood (Arita et al. 2006)	Dominant (Arita et al. 2006)
N14K	Abolished	Yes −80%	KIDS – survive to childhood (Lazic et al. 2008)	Dominant (Lazic et al. 2008)
A88S	Fully retained, but affinity shifted to higher PCO_2_	No	None – but only heterozygotes reported (Frei et al. 2002)	Recessive (Frei et al. 2002)
M34T	Greatly reduced, no effect on affinity for PCO_2_	No	Mild to Moderate NSHL (Snoeckx et al. 2005)	Recessive (Snoeckx et al. 2005)
V84L	Fully retained	Not tested	Profound NSHL (Kenna et al. 2001)	Recessive (Kenna et al. 2001)

DN, dominant negative; KIDS, keratitis ichthyosis deafness syndrome; NSHL, non‐syndromic sensorineural hearing loss.

Two further dominant mutations linked to KID syndrome, N14K, (van Steensel et al. 2004; Lazic et al. 2008) and N14Y (Arita et al. 2006) had effects on the CO_2_ sensitivity of Cx26 that were very similar to those of the A88V mutation (Table 1). Like Cx26^A88V^, Cx26^N14K^ can form functional gap junctions and hemichannels (Lee et al. 2009). We found that the CO_2_ sensitivity of Cx26^N14K^ was abolished, and this mutation had a powerful dominant negative action on the CO_2_ sensitivity of Cx26^WT^.

We were unable to express Cx26^N14Y^ in parental HeLa cells, but could do so in HeLa cells that stably express Cx26^WT^. It is possible that the wild‐type protein in some way chaperones the mutant subunit to enable expression. Thus, we do not know whether homomeric Cx26^N14Y^ retains CO_2_ sensitivity, but expression of this mutant subunit had a powerful dominant negative action on the wild‐type Cx26 and completely abolished CO_2_ sensitivity.

The clinical presentation of patients with A88V, N14K, and N14Y mutations differ. The A88V variant is particularly severe and patients die in infancy. Patients with the N14Y and N14K variants survive at least into childhood. The symptoms in individuals with the N14K mutation have been suggested to indicate a modified version of the syndrome (van Steensel et al. 2004; Lazic et al. 2008). By contrast, individuals with the N14Y mutation exhibit a more complete array of symptoms (Arita et al. 2006). In this regard, it is interesting and suggestive that the dominant negative action of N14K on CO_2_ sensitivity is not complete and leaves about 20% of the CO_2_ sensitivity in the wild type. By contrast, the dominant negative actions of N14Y and A88V (Meigh et al. 2014) are complete. A recent study has also compared the properties of the N14K and N14Y mutations on the gating of hemichannels and suggests that the N14K mutation may stabilize in the open state, whereas the N14Y mutation may do the opposite (Sanchez et al. 2016). We have not yet tested the effects of the six other KID syndrome mutations on CO_2_ sensitivity, so do not yet know whether the dominant negative action on CO_2_ sensitivity is common to all KID syndrome mutations.

**Figure 6:**
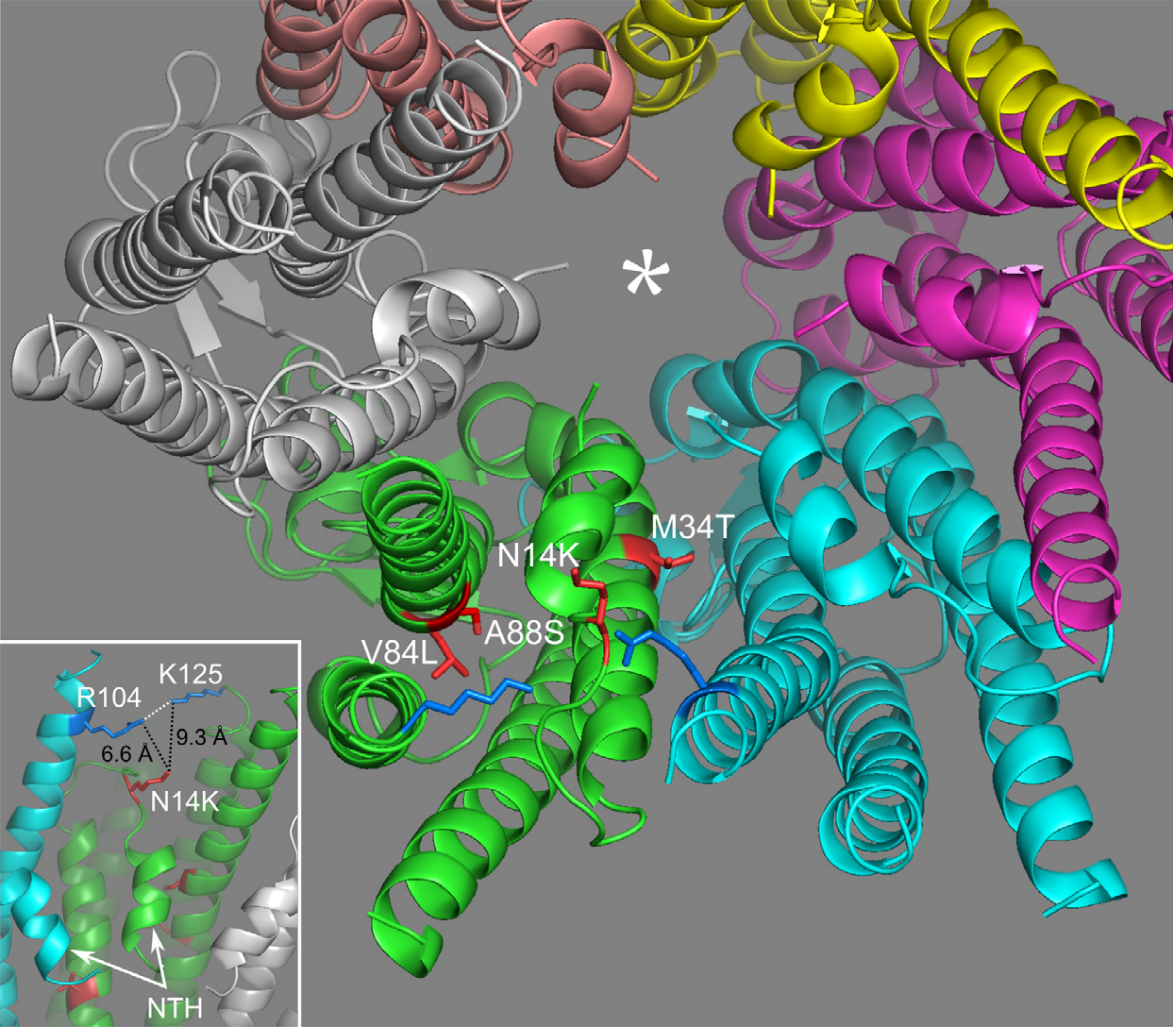
Locations of the mutations studied. The Cx26 hemichannel viewed from the cytoplasmic side along the axis of the central pore (*). In the green subunit, the mutated residues are shown in red. The residues in blue are K125, to which CO_2_ binds, and R104 of the neighboring subunit (to which the carbamylated lysine forms a salt bridge – dotted line, inset). V84 and A88 point into the interior of the subunit, whereas M34 points into the pore. N14 is present in the loop that connects the N‐terminal helix (NTH) to the rest of the subunit (inset). When mutated to lysine, this residue is within 10 Å of the CO_2_‐binding site (inset), and introduction of a charged residue at this location could plausibly alter the ability of CO_2_ to bind to Cx26. The mutation N14Y substitutes a bulkier aromatic tyrosine residue at the same location (not shown for clarity). Molecular model drawn from the PDB structure 2zw3.

Mutations at the N14 locus are situated in the cytoplasmic linker region that connects the N‐terminal alpha helix (present within the hemichannel pore) to the rest of the Cx26 subunit. Interestingly, the folding of Cx26 brings N14 rather close to the carbamylation site (K125) and the salt bridge acceptor (R104) of the neighboring subunit (Fig. 6). N14 is only ~10 Å from K125 and ~7 Å from R104. We speculate that the mutation of the polar asparagine residue to a positively charged lysine or a bulky tyrosine in the vicinity of the site of carbamylation could plausibly alter the environment around this site and hence cause the loss of CO_2_ sensitivity.

### Non‐syndromic deafness and CO_2_ sensitivity

We extended our studies to investigate mutations linked to non‐syndromic deafness. Although originally described as dominant (Kelsell et al. 1997), there are many who are heterozygous for this allele but who nevertheless have normal hearing (Scott et al. 1998). The M34T mutation only causes mild to moderate hearing loss when homozygous or in combination with other mutant alleles such as G35del (Snoeckx et al. 2005). This mutation did not appear to change the affinity of Cx26 for CO_2_ but did reduce the extent of dye loading presumably by interfering with the ability of CO_2_‐bound hemichannels to fully open. There was no dominant action of Cx26^M34T^ on the CO_2_ sensitivity of the wild‐type hemichannel. This is consistent with the observation that the M34T mutation has a recessive effect on deafness. However, the lack of dominant action of M34T on the CO_2_ sensitivity of Cx26 hemichannels contrasts with the possible dominant inhibitory action of this mutation on Cx26 gap junctions (D'Andrea et al. 2002; Bicego et al. 2006), although this dominant action has been contested (Skerrett et al. 2004).

The V84L mutation is another recessive mutation linked to hearing loss (Kelley et al. 1998). This mutation can cause profound hearing loss when homozygous (Kenna et al. 2001). HeLa cells coupled by Cx26^V84L^ gap junctions demonstrate reduced permeability to the Ca^2+^‐mobilizing messenger, inositol 1,4,5‐trisphosphate. This loss of metabolic coupling may be the mechanism by which this mutation causes hearing impairment (Beltramello et al. 2005). In contrast to all of the other mutations examined, we found that V84L had no effect on the CO_2_ sensitivity of Cx26. The V84 residue is in the same alpha helix as A88 (Fig. 6). There is thus a surprising contrast between the profound effects of the A88V mutation on CO_2_ sensitivity and the lack of effect of V84L. This observation highlights the need for better structural understanding of Cx26 so that we can understand the differing effects of these two mutations and A88S on the CO_2_ sensitivity of Cx26.

### Implications for etiology of KID syndrome

These findings and our previous publication show that the degree to which CO_2_‐sensitivity is lost from the mutant hemichannel has some relation to the emergent pathology in humans (Table 1). This is particularly true for the KID syndrome mutations where we have now tested three out of the nine known mutations that cause this syndrome and shown that these mutations also greatly reduce CO_2_ sensitivity of Cx26 hemichannels in a dominant manner. Interestingly, mutation of the A88 residue to serine, which does not cause KID syndrome, only has recessive and modest effects on CO_2_ sensitivity. The main hypothesis for the effects of the dominant missense mutations of Cx26 causing syndromes is that there is a gain of function – through increased hemichannel activity (Levit et al. 2014; Sanchez et al. 2014; Sanchez and Verselis 2014; Levit and White 2015; Lilly et al. 2016). Our data now provide an alternative or additional potential hypothesis – that the loss of CO_2_‐dependent modulation of hemichannel gating could contribute to pathology, at least for some mutations of Cx26.

Our original discovery of the effect of the A88V mutation on the CO_2_ sensitivity of Cx26 was stimulated by a KID syndrome patient carrying this mutation, who exhibited reduced respiratory drive (Meigh et al. 2014). Given the link between Cx26 and the chemosensory control of breathing that we have established in rodents (Huckstepp et al. 2010b), it seems possible that Cx26 hemichannels are also involved chemosensory control of breathing in humans. We can therefore make two important predictions from this study. Firstly, patients with N14K and N14Y KID syndrome, like those with the A88V KID syndrome, may also exhibit reduced respiratory drive. Secondly, individuals homozygous for M34T, or carrying this allele in combination with the common G35del allele, which is a truncating mutant that prevents expression of functional Cx26, should also be at risk of reduced chemosensory drive as the M34T mutation greatly reduces the ability of CO_2_ to open the hemichannel. Investigation of abnormalities in breathing in individuals with these mutations, or individuals who fail to express any functional Cx26 (e.g., homozygous for G35del) could be both highly informative and potentially beneficial in a clinical setting.

## Conflict of Interest

The authors declare that there are no conflicts of interests.
